# Urban Indian young adults’ perceptions of a healthy lifestyle and its associated factors: a multi-city qualitative inquiry using thematic analysis

**DOI:** 10.3389/fpubh.2026.1886704

**Published:** 2026-07-08

**Authors:** Neha Rathi, Aditi Sharma, Shreya Sandilya, Sumana Ghosh, Reshab Chowdhury, Mukta Singh, Anthony Worsley, Meg Bruening

**Affiliations:** 1 Bagchi School of Public Health, Ahmedabad University, Ahmedabad, Gujarat, India; 2 Department of Home Science, Mahila Mahavidyalaya, Banaras Hindu University, Varanasi, Uttar Pradesh, India; 3 School of Nutrition and Dietetics, Symbiosis Skills and Professional University, Pimpri-Chinchwad, Maharashtra, India; 4 School of Exercise and Nutrition Sciences, Deakin University, Geelong, VIC, Australia; 5 Department of Nutritional Sciences, College of Health and Human Development, The Pennsylvania State University, University Park, PA, United States

**Keywords:** health, India, interviews, lifestyle, qualitative, young adults

## Abstract

**Introduction:**

The prevalence of overweight and obesity and the associated non-communicable diseases in young adulthood has increased significantly in the last decade. Urban Indian young adults fail to meet the recommended guidelines for healthy diet and physical activity. The aim of this qualitative study was to gain novel insights into the barriers and facilitators of a healthy lifestyle of Indian young adults residing in urban settings.

**Methods:**

Underpinned by the social constructivism paradigm, in-depth interviews were conducted with young adults (aged 21–30 years) who were recruited using purposive and snowball sampling techniques from eight metropolitan cities (New Delhi, Mumbai, Kolkata, Chennai, Ahmedabad, Pune, Hyderabad, Bengaluru). Interviews were audio recorded and transcribed verbatim. Transcripts were transferred to NVivo software for inductive thematic analysis.

**Results:**

A total of 79 young adults (41 males, 38 females) participated in the interviews. Five themes and their associated sub-themes were identified: (i) Perceptions of a healthy lifestyle (consuming a balanced diet, leading an active life, having adequate sleep); (ii) Facilitators of a healthy lifestyle (family support and upbringing, health promotion by celebrities and famous personalities, built environment, body image, intrinsic motivation); (iii) Barriers to a healthy lifestyle (time constraints, food environment challenges, peer pressure); (iv) Strategies adopted to maintain a healthy lifestyle (dietary management, engaging in exercise and other forms of physical activity, reducing screen time); and (v) Recommendations for prospective interventions (education and awareness about nutrition and health, creating healthy and hygienic food environments).

**Conclusion:**

The evidence emerging from this multisite inquiry offers first-hand information about the individual, social, and environmental level barriers and facilitators to the adoption of a healthy lifestyle among urban Indian young adults. Strategies and recommendations suggested during the interviews can be used to tailor prospective lifestyle interventions for encouraging the adoption of sustainable healthy lifestyle in young adults. Prospective interventions could focus on enhancing nutrition and health knowledge of young consumers and offering more healthy and hygienic food options in cafeterias of workplaces and educational institutions as well as other eateries.

## Introduction

India is home to one of the largest cohorts of young adults globally, with about 65% of its population below 35 years of age (1). Young adulthood is a unique developmental life phase that occurs between adolescence and adulthood (2). This transitional period is characterized by various life-changing events which can involve leaving the family home and school for pursuing higher education or entering the labor force, and establishing partner relationships including marriage, resulting in cohabitation (3–7). Besides these life-changing episodes, young adults’ engagement in risk-taking behaviors and unhealthy lifestyle make them an especially vulnerable population (2).

Some common unhealthy behaviors exhibited by young adults both in India and overseas include frequent consumption of energy-dense, nutrient-poor food and sugar-sweetened beverages, limited consumption of fruits and vegetables, having a sedentary lifestyle, use of tobacco and heavy drinking (8–12). These behavioral choices have resulted in the rising prevalence of overweight and obesity (13–15) and its associated chronic degenerative ailments including diabetes mellitus (16, 17) and hypertension (18, 19) in young adults. These health complications might continue and could further aggravate during middle and late adulthood (20).

The mean percentage of body mass index (BMI) has increased considerably over the last two decades, especially among young adults (2, 13, 14, 21, 22). A cross-sectional study from Mumbai reported that 42% of the respondents (*N* = 269; 18–24 years) had obesity (13). In addition, the high diabetes burden among Indian youth is apparent in a nationwide screening campaign wherein 13.3% of the participants (*N* = 27,925) below 30 years of age were found to be diabetic (17). Similarly, the prevalence of prehypertension and hypertension among young adults (*N* = 1,221) from the southern state of Kerala was 33.3 and 11.2%, respectively (19).

Unhealthy behaviors that develop in young adulthood often continue into middle and older adulthood, resulting in detrimental health outcomes (2, 23). This calls for the development and implementation of health-promoting lifestyle interventions to modify the unhealthy lifestyle choices of young people, thereby reducing the chronic degenerative disease burden (2, 10, 24).

The World Health Organization (WHO) describes the term ‘healthy lifestyle’ as a way of living that diminishes the risk of severe morbidity or premature mortality (25). A number of social and behavioral frameworks (26, 27) have been employed to explain and promote healthy lifestyle which include Health Belief Model (HBM) (28), Theory of Planned Behavior (TPB) (29), Transtheoretical Model (TTM) (30), Social Cognitive Theory (SCT) (31), and Socio Ecological Model (SEM) (32, 33). These theoretical models have been effective in highlighting the various determinants (i.e., both facilitators and barriers) of health behaviors at different levels such as individual (HBM, TPB), interpersonal level (SCT, TTM) and ecological level (SEM) (26). Likely determinants of healthy lifestyle at the individual level include food and health literacy, motivation, subjective norms, time, age, and gender (26, 34–36). At the interpersonal level, family and peers have shown to impact lifestyle through social support (26, 34). The availability and accessibility of food in educational institutions and workplace, open gymnasiums, retail food environments in residential neighborhoods, policies and taxes, lobbying and powerful marketing by large food corporate giants are commonly recognized ecological influences of lifestyle (26, 34, 36–38). These determinants may either facilitate or encumber healthy lifestyle (26, 34).

Although empirical evidence on the facilitators and barriers of healthy lifestyle is expanding globally, there is scant evidence available about the situation among Indian adults’ contexts. Moreover, the limited number of Indian studies have primarily focused either on dietary behaviors or physical activity in primary school children, adolescents, middle-aged and older adults as well as patients suffering from non-communicable diseases (39–42). In addition, there is paucity of local qualitative studies on healthy lifestyles. Only two local quantitative studies (10, 24) have assessed the health-promoting lifestyles of college students. Therefore, there is very limited information about young Indian adults’ perceptions of healthy lifestyle and their associated factors, or more generally among young people in the Global South. The use of qualitative research methods, then, is warranted in the present study because in-depth, qualitative interviews allow for a deeper understanding of healthy and unhealthy food behaviors, encapsulating a diversity of perspectives held by young Indian adults.

Therefore, the aim of the present study was to identify key perceptions of young Indian adults about healthy lifestyles and their associated factors using in-depth, qualitative interviews. Findings from this qualitative study have the potential to design targeted interventions to mitigate the barriers and enhance the facilitators for promoting healthy lifestyles and thus preventing the escalation of chronic degenerative diseases (43, 44).

## Methods

### Study design

Guided by the social constructivism paradigm (45), an interpretive, qualitative, descriptive research approach (46) was used in this study. Social constructivism, a phenomenological framework, assisted in elucidating respondents’ (i.e., young adults) understanding of the subject matter under investigation (i.e., factors influencing the adoption of a healthy lifestyle). Accordingly, the perspectives of the respondents are proposed to be formed through their individual experiences and social exchanges with other members of society (47). The implementation of this phenomenological framework is viable in the current context as the primary research objective was to assess how young adults perceive healthy lifestyle and various determinants that are either facilitators or barriers in the adoption of a healthy lifestyle in urban Indian settings. Qualitative data were collected using in-depth interviews (48). The close alignment between social constructivist epistemology, interpretive approach, qualitative descriptive methodology, and in-depth interviews facilitated the attainment of internal coherence in this inquiry (46).

To further enhance the methodological rigor of the study, a 32-item Consolidated Criteria for Reporting Qualitative Research (COREQ) checklist (49) was utilized to ensure robust reporting of all methodological aspects (i.e., research team, research design, data collection and analysis) of this study (See Supplementary file). The research protocol for this study received ethical approval (IMS/IEC/29) from the Ethics Committee of Banaras Hindu University.

### Research team

Six female (NR, AS, SS, SG, MS, MB) and two male investigators (RC, AW) formed the research team. NR is a mid-career scientist specializing in food behavior and qualitative research techniques. AS, SS, SG, and RC are postgraduate students who received prior training in conducting in-depth interviews and thematic analysis. Both MS and MB are senior professors of home economics and public health nutrition, respectively. AW is a retired professor of health psychology with broad experience in mixed-method and policy research. This alliance of investigators from different academic disciplines minimized the possibility of any personal, gender or professional biases incurred during the process of data interpretation.

### Study setting and sample recruitment

Young adults aged 21–30 years living in urban Indian settings comprised the study sample. Overweight and obesity as well as hypertension and diabetes are more prevalent in urban Indian youth when compared to their rural counterparts (50–54), thus validating our selection of young adults from Indian cities. Purposive sampling coupled with snowball sampling was used to recruit young adults from eight Indian metropolitan cities. Initially, study respondents were recruited through purposive sampling. Subsequently, few interviewees referred other young adults who could provide additional perspectives. Complementing purposive sampling, snowball sampling technique helped facilitate sample recruitment, improve access to eligible participants within the study setting, and improved the diversity of lived experiences within the study sample. At present, India has eight metropolitan cities (i.e., cities which have a population of four million or more) including New Delhi, Mumbai, Kolkata, Chennai, Ahmedabad, Pune, Hyderabad and Bengaluru (55). These eight cities were chosen for this inquiry because of their dynamic urban settings, fast-paced life and rich cultural diversity, factors that typically influence the lifestyle of young people. Moreover, considering the cosmopolitan nature of these cities, the study respondents were likely to represent India’s urban young adult demographic rather than young adults from rural areas.

Young adults were recruited via multiple social media platforms, such as Facebook, Instagram, WhatsApp and LinkedIn. Interested respondents were subsequently given a consent form and a plain language statement describing the research topic and interview process. Upon receiving written consent, respondents were required to indicate their preferences regarding the interview’s time, date, mode (in-person or virtual), and location (for in-person interviews). In the context of qualitative research, virtual interviews (either conducted over telephone or online platforms like Zoom or Google Meet) are widely recognized as valuable substitutes to physical (in-person) interviews because the quality of data remains unaltered irrespective of the method executed (56–59).

### Interview guide

Underpinned by the research objective and previous scholarship on healthy lifestyles (34) an interview guide comprising seven open-ended questions was developed (Table 1). NR pre-tested the interview guide with four young adults (i.e., 2 males and 2 females), all four of them were able to reply effectively to the proposed questions. Therefore, no alterations were deemed necessary. In addition to the open-ended questions, socio-demographic information including age, educational qualification, monthly income, employment status and marital status was also gathered from the respondents.

**Table 1:** Interview guide.

Question No.	Open-ended questions
Q1	In general, what do you think the phrase “healthy lifestyle” means?
Q2	Personally, what does a healthy lifestyle mean to you?
Q3	Can you talk about some of the factors which influence the adoption of a healthy lifestyle?
Q4	Among the factors highlighted by you, are there any barriers or challenges to adopting a healthy lifestyle?
Q5	How do you overcome these challenges?
Q6	Among the factors you just mentioned, are there any facilitators that enable one to lead a healthy lifestyle?
Q7	Can you think of any initiatives/schemes that can be implemented in your locality to make it easier for young adults to lead a healthy lifestyle? What would you like to see implemented in the future?

### Data collection

Taking into account respondents’ preferences, interviews were conducted either virtually (i.e., telephone/online over Google Meet) or face-to-face in English or Hindi. In the presence of a note keeper, the interviews were conducted by postgraduate students (AS/SS/SG/RC) who had received training in qualitative data collection techniques and analysis from the lead investigator (NR). All the four interviewers as well as NR are fluent in both English and Hindi. The conversations were audio recorded. Interviews were conducted until thematic saturation was achieved, with no new concepts/ideas emerging during the interviews (60). Inductive coding was performed simultaneously with the interview sessions to actively check for saturation during fieldwork. Saturation was attained at the 79th interview as no new codes appeared across the last five interviews. Interviews ranged from 10 min to 42 min.

### Data analysis

A denaturalized technique (i.e., focusing on interview material while deleting interview noise like pauses and stutters) was used for transcription of interview recordings (61). All the interviewers transcribed their recordings verbatim and translated to English where necessary. NR cross-checked 25% of the total transcripts with the digital recordings to ensure accuracy. To enhance the credibility of our findings, member checking (i.e., participant validation) was performed whereby the transcripts were returned to the original respondents to confirm accuracy of the transcribed data (62, 63). Only six respondents engaged in member checking and none of them requested any modifications in their transcripts. Interview transcripts were imported to the NVivo 15 software program for open coding.

A six-phase analytical process was adopted for inductive thematic analysis (64, 65). In the first phase, the researcher read and re-read the entire dataset to facilitate a deep immersion into the interview data. In the next phase, codes, the fundamental building blocks of the themes were generated through open coding. The third phase encompassed reviewing and analyzing the initial codes (i.e., collapsing multiple codes according to shared meanings) to form major and minor themes. Consequently, a recursive assessment of the potential themes in connection with the coded items and the complete dataset was carried out. In the second last phase, the coder defined and named the theme by providing detailed descriptions to improve clarity. Finally, a comprehensive report including a detailed account of themes, supporting quotations, and analytic commentary was prepared (64–66).

Open coding was performed by two coders. The lead author (NR) coded the entire data set while the second coder (AS) coded 50% of the interview transcripts to enhance reliability and minimize any subjective bias. In case of any discrepancies between the two coders, a third coder (SS) was consulted for resolution. The final codes were reviewed by all the authors.

## Results

Seventy-nine young adults (48% female) participated in the in-depth interviews. A little more than half of the sample was living with their parents and were employed. The mean age of the participants was 24.30 years (SD: 2.62) and two-thirds of the sample had attained an undergraduate degree (Table 2).

**Table 2:** Demographic characteristics of study participants (*N* = 79).

Characteristics	*N* (%)
Gender	
Female	38 (48)
Male	41 (52)
Education	
High school	5 (6)
Bachelors	53 (67)
Masters	19 (24)
Doctorate	2 (3)
Living arrangement	
“Parental household” (living with family)	43 (54)
“Independent household/hostel” (not living with family)	36 (46)
Student status	
Currently studying	19 (24)
Not studying	60 (76)
Employment status	
Employed	42 (53)
Unemployed (includes unemployed students)	37 (47)

A number of themes and sub-themes associated with adoption of a healthy lifestyle were identified during the thematic analysis of the interview transcripts which included: (i) Perceptions of a healthy lifestyle; (ii) Facilitators of a healthy lifestyle; (iii) Barriers to a healthy lifestyle; (iv) Strategies adopted to maintain a healthy lifestyle; and (v) Recommendations for prospective interventions (Table 3). These themes and their associated sub-themes along with illustrative quotations are described below:

**Table 3:** Themes and sub-themes associated with adoption of a healthy lifestyle.

Themes	Sub-themes	Quotes
Perceptions of a healthy lifestyle	Consuming a balanced diet	*“…food being sold in a street corner… that is completely unhealthy for me. I consider that as… unhealthy diet. For me you can say… uh… a bit light food… less spicy… less oily… homemade food…”* (M32, 24 years, Kolkata) *“Avoid unwanted things like unhealthy food and stress so that you can also avoid some diseases too”* (F37, 22 years, Pune)
	Leading an active life	*“…so… uh… yeah… I do make sure… you know… to go to Zumba… and uh… after I wrap up my work… I… most of the days I try to go to the gym…”* (F16, 28 years, Mumbai) *“I go to the gym, and I go to the park to exercise regularly for many years. So, I try to do physical exercise so that my body is in good shape”* (M3, 22 years, New Delhi)
	Having adequate sleep	*“Speaking of a healthy lifestyle, what comes to my mind is maintaining a proper sleep cycle and sleeping on time…”* (F22, 25 years, Kolkata) *“Inadequate sleep is a problem…”* (M34, 24 years, Kolkata)
Facilitators of a healthy lifestyle	Family support and upbringing	*“In my family, my brother is the one who is maintaining a strict diet. I just try to follow him like whatever he does…”* (M11, 29 years, Chennai) *“In my house, like my mother-in-law, she is very conscious when it comes to her health. In fact, she motivates me all the time to do the right kind of things… Now I have started doing Yoga after seeing my mother-in-law.”* (F34, 25 years, Pune)
	Health promotion by celebrities and famous personalities	*“…I’ve seen on international yoga day that the Prime Minister himself does yoga and promotes it. Then I also saw our Sports Minister doing a workout and his video went viral in 23* (2023) *in which he is promoting it and asking everyone to indulge in workout to stay healthy…”* (F3, 23 years, New Delhi) *“…like Shilpa Shetty* (Bollywood actress) *has a very healthy lifestyle, I used to see. I learned from her to like to eat healthy food like there is no need to cut like what I say to kill your desires, to kill your cravings…”* (F34, 25 years, Pune)
	Built environment	*“Like now, I have started doing running and some basic exercise. Freestyle exercise, stretching. And nearby we also have a community center. There are some gym exercises and equipment available. So, I go for that. That includes cycling and stretching.”* (F5, 22 years, New Delhi) *“If someone wants to play anything to maintain a healthy lifestyle… They can play a lot of sports like cricket, uh… badminton, etc. A lot of playgrounds are located in my area as well…” (F24, 26 years, Kolkata)*
	Body image	*“Like, because I think that I want to look good in good clothes. So, I have to maintain my diet as well as my body. So, I would definitely go for a good diet…”* (F4, 23 years, New Delhi, Female) *“Gym gives me self-motivation to look good—otherwise my belly will pop out. Just think about whether you want to have cervical pain at the age of 40… This is my main motivation.”* (M2, 28 years, New Delhi)
	Intrinsic motivation	*“Nothing is impossible! I know some men who work 12 h, and they still come to the gym after office hours. If you have the motivation or energy, you will go to the gym or do something for your health… Lethargy are all excuses…”* (F23, 23 years, Kolkata) *“The matter of fitness or adhering to a healthy diet is a matter of inner motivation.”* (M28, 25 years, Kolkata)
Barriers to a healthy lifestyle	Time constraints	*“So, if I talk about specifically for my company, there is no dedicated timing for the meal. So, it is like you have certain meetings, you have certain deliverables and you have nine to ten hours of work…”* (F1,29 years, New Delhi) *“There are challenges like you have to come and make the food yourself or help in making it, so it takes a lot of time. And I do not even feel like making it sometimes*. *There are times when I cannot make it so I have to eat outside or I opt for a substitute which can be made easily*.*”* (M4, 21 years, New Delhi)
	Food environment challenges	*“Junk food is…since junk food is much tastier and they are available easily*. *So, for that reason… we may end up consuming more junk food…”* (F25, 29 years, Kolkata) *“Zomato will deliver the food within ten minutes*. *BlinkIt will deliver snacks within ten minutes*. *Just open the packet and eat your food*. *It was not like this in earlier days…The earlier generation used to prepare good food at home*. *There were not so many food chains or restaurants in Kolkata in the earlier days…Now there are a number of people who are addicted to fast food and junk food*. *I am one of them*. *I do not deny it…”* (M27, 21 years, Kolkata)
	Peer pressure	*“I have few friends, let us say everybody is going to have cold coffee today*. *Now I cannot cancel that and go have buttermilk*. *I also have to order cold coffee with them!”* (F32, 21 years, Pune) *“If you go out with friends and everyone is eating fast food, you feel odd saying no*. *You just go with the flow even if you know it’s not good for your health!”* (M6, 25 years, New Delhi)
Strategies adopted to maintain a healthy lifestyle	Dietary management	*“… I prefer light and healthy options—like beet juice, dried fruits, or something easy to digest”* (F34, 25 years, Pune) *“As much as possible… uh… I try to eat a low-carb diet… and have a high protein intake*. *Uh… I try to have chicken for at least one meal of the day… every day*. *That is, it!”* (M33, 21 years, Kolkata)
	Engage in exercise and other forms of physical activity	*“I try to walk whenever I can—like if the grocery store is within a kilometer, I will not take the bike*. *These small things add up*.*”* (M11, 29 years, Chennai) *“…I’d say… in my own case… while on my way back home from the university… many times… I get off the bus at some distance from my home… and from there, I come home walking…”* (M27, 21 years, Kolkata) *“After brushing my teeth, I drink lukewarm water and go for a walk*. *After walking, I do some exercise*.*”* (F13, 22 years, Chennai)
	Reducing screen time	*“Most of the time, people are occupied with their laptops and phones, making it difficult to focus on physical well-being*. *That’s why I believe a digital detox is essential… reducing screen time is crucial for mental well-being*.*”* (M2, 28 years, Delhi) *“Screen time also affects the sleep cycle in a major way… Personally I have a problem that is I get a headache if I watch the mobile screen for too long*. *For that reason, I consciously avoid it and try to regulate my screen time…”* (F22, 25 years, Kolkata)
Recommendations for prospective lifestyle interventions	Education and awareness about nutrition and health	*“I think that people should eat as much simple food as possible*. *They should eat less spicy and oily food*. *Because I think that oily food affects the body*.*”* (F11, 21 years, Chennai) *“What I feel is that… it could make things easier if, for example… uh… if this subject of nutrition could be made mandatory in our schools and colleges…”* (F22, 25 years, Kolkata)
	Creating healthy and hygienic food environments	*“See we need to stop the supply of junk food in offices*. *Then only people will stop eating such stuff*. *Moreover, office canteens should sell healthy food instead…”* (F35, 22 years, Pune) *“…in my office I do not see many healthy food options*. *They can supply some healthy food like rice, dal, vegetables…”* (M28, 25 years, Kolkata)

### Perceptions of a healthy lifestyle

#### Consuming a balanced diet (54/79)

Across all locations and genders, the interviewees perceived that a balanced diet laid the foundation for a healthy lifestyle. A balanced diet was consistently described by participants as fresh, home-cooked, nutritionally adequate, less oily and spicy, as well as free from excessive processing. Participants associated consumption of a balanced diet not only with physical health benefits but also with self-control, discipline, and long-term disease prevention. They further highlighted the importance of moderation and portion control rather than extreme dietary restrictions.

*“A healthy lifestyle means you could say eating fresh food, avoiding all sorts of junk food, and if we avoid outside food, then with that we can lead a healthy lifestyle…”* (F21, 27 years, Kolkata).

*“Speaking of a healthy lifestyle, the first thing that comes to my mind is diet control*. *With a balanced intake of protein, carbohydrates, and fat…”* (M24, 29 years, Kolkata).

#### Leading an active life (52/79)

Engaging in regular physical activity such as walking, yoga, gym workouts, sports, or stretching was recognized as an integral component of a healthy lifestyle. Physical movement was viewed not only as a way to maintain physical fitness and endurance but also as a requisite for stress relief and mental well-being. The study participants often emphasized consistency more than intensity. Some of them also noted that physical movement was necessary even if eating habits fluctuated. Exercise was conceptualized broadly and included both structured (e.g., gym workouts, yoga) and unstructured (e.g., walking to a nearby grocery store, climbing stairs) activities.

*“Uh, well, some people talk about exercising (daily) for an hour or so…I, too, feel the same*. *If not for an hour, then at least for half an hour daily, if someone practices Yoga like I personally practice Yoga…I mean I try, if I am not all too busy, then I try to do it (daily), otherwise there’s walking…”* (F20, 25 years, Kolkata)

“*Healthy lifestyle is doing some exercise everyday…Rather than taking the lift in my office, I choose to climb the stairs*.” (M2, 28 years, New Delhi)

#### Having adequate sleep (34/79)

Another important element of a healthy lifestyle perceived by our participants was having adequate sleep. According to the participants, a healthy sleep regime comprised seven to eight hours of sleep per night. They also reported that going to sleep at the right time was also a critical part of a healthy lifestyle. Some noted that sleeping at odd times or having insufficient sleep had negative implications for both physical and mental health. In addition, two interviewees attributed insufficient sleep to excessive use of mobile phones for entertainment purposes during late night.

*“In general, a healthy lifestyle for me is taking your meals regularly from time to time; proper healthy meals obviously…and at least eight hours of sleep, that’s it…and also pursuing an active lifestyle*. *That’s what I understand by having a healthy lifestyle… sleep time is really important, otherwise it is not possible to function properly*.*”* (F20, 25 years, Kolkata)

*“Sleep…I think one should sleep for eight hours and if that’s not possible then six hours definitely…”* (M21, 23 years, Hyderabad)

### Facilitators of a healthy lifestyle

#### Family support and upbringing (42/79)

The interviewees viewed upbringing and family support as prominent influences in facilitating and sustaining a healthy lifestyle. They acknowledged that early and repeated exposures to healthy lifestyle in their homes established a robust foundation for leading a disciplined lifestyle as well as contributed to the development of lifelong habits. Young adults also believed that their family members served as positive role models for healthy lifestyle.

*“…My maternal grandfather is above 80, above 85 actually*. *He is more active than us… He does yoga daily*. *He wakes up at 5-6 o'clock in the morning*. *So, I look up to him when it comes to exercising!” (M6, 25 years, New Delhi)*

They further appreciated the constant emotional support (i.e., words of reassurance that offered relief) rendered by their family members, particularly during periods of stress or diminished intrinsic motivation. This emotional reinforcement played a crucial role in sustaining engagement with health-promoting lifestyle among our interviewees as illustrated below:

*“…I mean, they (parents) too must be saying it for my well-being only… I was already an oversized kid, I mean overweight kid*. *I was ninety when I was in my twelfth class*. *So… from there, now I am seventy*. *So… that particular change has somehow happened in a good way… only because of my family members…They have been highly supportive in my weight loss journey…” (F23, 23 years, Kolkata)*

#### Health promotion by celebrities and famous personalities (41/79)

Health promotion by famous personalities including athletes, fitness influencers, public figures (e.g., a politician, television actor) on social media and television was often cited as a major facilitator in boosting the young adult participants to adopt and maintain a healthy lifestyle. The celebrities/famous personalities were viewed as role models for both disciplined lifestyle and physical appearance. Participants noted that observing the journeys of these personalities helped them remain motivated during episodes of low enthusiasm, plateaus, or relapse, reinforcing adherence to healthy lifestyle over time. The participants further highlighted that endorsements and health advocacy from prominent personalities enhanced the integrity and mass appeal of health-related messages, thereby making healthy lifestyle easily attainable and socially acceptable.

*“Other than this when I see a cricketer like Virat Kohli, that at the age of 40 he is in such good shape then I can also do it*.*”* (M5, 21 years, New Delhi)

*“Social media influencers who promote healthy lifestyles*. *For example, from Hyderabad if you see Navin golden boy, he's one of the popular social media influencers who shares tips on how to remain fit and what all we can include in our diet…”* (M20, 25 years, Hyderabad)

#### Built environment (44/79)

A recurring facilitator for adopting and sustaining healthy lifestyle referred to during the participants’ interviews was the built environment. Many of our participants acknowledged the presence of both public and private gymnasiums, public parks as well as walking tracks either in their residential neighborhoods or workplaces. These built-in infrastructures offered easily accessible opportunities to our participants for regular engagement with physical activity as noted below:

*“Yeah, so they have many courts*. *We have a badminton court and volleyball court… So, I play all these sports in my office campus only*.*”* (M8, 30 years, New Delhi)

*“There are a lot of open-air gyms here*. *And there’s a ground where people run and exercise…”* (F38, 27 years, Ahmedabad)

Additionally, some of the interviewees also talked about participating in marathons which not only boosted their physical fitness but also contributed to the development of social connections, subsequently enhancing their social well-being.

*“There are weekend marathons in Delhi… you have to run in the morning, and not only do you get healthy, but you also get to know other people—what they are capable of, your colleagues. It’s a type of bond-building between your colleagues and people from other companies as well.”* (M1, 23 years, New Delhi)

In contrast to the promising insights about the built environment, two of the interviewees lamented that there were no gyms or public parks in their vicinity for doing exercises. Those who had open gyms in their neighborhood also could not utilize the public facility for exercising as the majority of the gym machines were not functional, as indicated by one of our interviewees:

*“There is an open gym in the park near my place, but most of the machines are broken. No one really uses them now.”* (M16, 25 years, Mumbai)

#### Body image (44/79)

For the majority of our interviewees, maintaining a good body shape and weight was an important facilitator for adopting a healthy lifestyle. They believed that eating nutritious food and performing breathing exercises impacted their physical appearance in a positive manner. Many aspired to remain fit, lean, and maintain a certain aesthetic standard which in turn assisted them in navigating societal expectations. Their motivation to appear confident and good appeared to be a strong influence in prompting some of our participants to abandon their existing unhealthy habits.

*“The more you do breathing exercises, the more your skin will glow. Like, I feel like that.”* (F12, 23 years, Chennai)

*“…now why do I go to the gym? To look good. At the end of the day… What is the conclusion? What is the moral of it all? What is my priority? I want to look good… and for that… I need to do so many things…”* (M32, 24 years, Kolkata)

#### Intrinsic motivation (47/79)

Another important facilitator for the establishment and preservation of healthy lifestyle identified during the interviews was individual motivation. The participants strongly believed that motivation must come from within oneself rather than external pressures to adopt and sustain healthy behavior. Along with motivation, a firm internal conviction was also essential to drive a positive behavior change. For some interviewees, the journey towards health and well-being commenced with a personal realization grounded in self-determination, often triggered by concerns related to body weight and future health outcomes.

*“It all depends on me. I go out with my friends, but if I don’t want to eat something, I don’t eat!… If you eat with your friends, it’s fine; if you don’t, it’s fine too.”* (M41, 25 years, Ahmedabad)

*“Secondly… motivation must be there. Without the self-motivation… nothing is possible. Self-motivation and the will power should be there within a person…”* (F29, 24 years, Kolkata)

*“I go to the gym and exercise regularly… so that when I age, I don't have any health-related problems.”* (M3, 22 years, New Delhi)

### Barriers to a healthy lifestyle

#### Time constraints (57/79)

The most commonly cited barrier to adopting a healthy lifestyle during the interviews was time constraints. All the participants expressed their frustration about not maintaining healthy behaviors because of lack of time. They attributed this barrier either to long work hours in their workplace or to excessive academic load which resulted in exhaustion. Subsequently the respondents were left with no stamina to either exercise or cook healthy meals for themselves. Some even complained about erratic work or academic (class timetable) schedules which further inhibited the respondents from following a healthy lifestyle.

*“I want to adopt a healthy lifestyle, but my problem is… somewhat like I have to manage my work and even my healthy diet, and everything according to that. But it doesn’t happen in the same way because uh… maybe (because) I have to work overtime…” (F29, 24 years, Kolkata)*

*I don't have time because of the lack of work-life balance. Plus, I travel a lot. So, due to long travel and lack of time, I end up doing nothing. (M16, 25 years, Mumbai)*

#### Food environment challenges (52/79)

Another frequently cited set of barriers to maintaining a healthy lifestyle were food environment challenges. The food environments inside the workplace or academic institutes were criticized for selling energy-dense, nutrient-poor foods and sugary drinks. Likewise, the neighborhood food environment including restaurants and fast food eateries was also devoid of healthy food options.

*“If you're working in a company and there’s no healthy cafeteria and you're hungry, what will you do? Will you starve all day or just eat whatever is available? So, I guess that’s what used to happen to me in college too…”* (F34, 25 years, Pune)

Another recurring opinion was that food delivery applications like Zomato, Swiggy, UberEats promoted fast food over healthy food like soups and salads by offering huge discounts over pizza, burgers, and fries as highlighted in the following quote:

*“Junk food is promoted much more on these apps compared to healthy food. As a result, people are tempted to order junk food, especially when they see discounts and offers. Since healthy food isn’t promoted as much, it doesn’t get the same attention.” (M3, 22 years, New Delhi)*

#### Peer pressure (40/79)

Peer pressure was often identified as a major deterrent to practicing healthy lifestyle. Some participants felt compelled to conform to peer norms to avoid standing out or being judged negatively, particularly in group settings in which indulging in unhealthy eating or skipping physical activity was normalized. They even criticized their peers for subtly downplaying the importance of healthy eating or making healthy eating seem unwarranted.

*“If I go out with my friends (They would say), ‘You can eat pizza for one day…nothing will happen’…”* (F23, 23 years, Kolkata)

*“So, it depends if he's eating a pastry or a cake. Then I also have to join him, because if I do not join him, then it may leave a negative impression on his mind that this person is different.”* (M1, 23 years, New Delhi)

### Strategies adopted to maintain a healthy lifestyle

#### Dietary management (49/79)

One popular strategy adopted by the study participants for maintaining a healthy lifestyle was swapping ultra-processed foods with minimally processed foods. Some participants mentioned reducing their episodes of unhealthy snacking. Likewise, they also discussed cooking meals at home and consuming them. The participants perceived that home cooking allowed better control over ingredients, portion sizes, and overall calorie intake. Preference for a high-protein diet was also highlighted in a couple of interviews.

*That wasn’t healthy, but once we shifted to a flat and everything was set up—like having a cook who prepared proper meals—it became much better. Now, we have more control over what we eat…”* (M13, 23 years, Chennai)

*“So, food wise, I try to follow a healthy lifestyle. For example, in breakfast, generally, I have three to four options. Mostly, I prepare poha* (rice flakes sautéed with vegetables) *or boiled egg, or I go for boiled egg and banana shake or poha and banana shake…”* (F17, 29 years, Bengaluru)

#### Engaging in exercise and other forms of physical activity (52/79)

In order to have a healthy lifestyle, nearly two-thirds of the sample engaged in physical activity on a regular basis. They either went to yoga classes or gymnasiums to have an active lifestyle, and this became a part of their daily routine. Creating a structured schedule especially in relation to walking or exercising supported the young adults in making it a sustainable and consistent activity. They believed that structured physical activity schedules provided clear goals which constantly stimulated them to lead an active lifestyle.

*“Following a (particular) routine could be difficult for one or two days…But if one dedicatedly sticks to that routine, then after a few days…automatically it will become easy…”* (F28, 22 years, Kolkata)

*“It was like I was not happy with myself…So, I just started taking little steps and slowly I started seeing or witnessing the changes and the progress and it motivates me a lot and then it just took me one year and then I think I have lost 25 kgs and I am like yeah it was great like definitely. It's not just about losing weight, it's about exercising every single day…”* (F3, 23 years, New Delhi)

Perseverance to stay physically active enabled the participants to gradually advance towards their individual health goals as well as sustain long-term health and well-being as reflected in the following quotation:

*“I go to the gym and exercise regularly… so that when I age, I won't have any health-related problems in the future.” (M3, 22 years, New Delhi)*

On the other hand, there were some participants who had a very hectic or an unstructured academic/work schedule and could not participate in structured physical activity sessions. Nevertheless, these participants tried to incorporate regular movement in their daily regime either by using stairs instead of elevators or walking to a nearby grocery store instead of using a motorbike as narrated below:

*“Rather than taking the lift in my office, I choose to climb the stairs.*” (M2, 28 years, Delhi, Male)

For interviewees with desk jobs, substituting prolonged sitting time with short bouts of physical movement like stretching, standing during phone calls, or brief walks to the water station were viable approaches to counteract the adverse health effects of sedentary living as suggested by one of the male interviewees:

*“After every hour of sitting, I get up and walk around for 5 minutes. It clears my head and also helps me avoid back pain.”* (M20, 25 years, Hyderabad)

#### Reducing screen time (39/79)

A small proportion of the sample talked about willfully reducing the time spent on online games and social media for their mental well-being. The participants mentioned that excessive usage of smart-phones hampered their sleep regime and even caused headaches and therefore they either tried to uninstall the game applications from their phones or stopped browsing social media handles one hour prior to going to bed. These views are illustrated in the following two excerpts:

*“I keep my phone away an hour before sleeping—otherwise I just keep scrolling and sleep late, which spoils everything the next day.”* (M5, 21 years, New Delhi)

*“If my head is reeling then what I do is that I uninstall that game…”* (F28, 22 years, Kolkata)

### Recommendations for prospective interventions

#### Education and awareness about nutrition and health (55/79)

One of the most frequently cited recommendations for adopting and sustaining a healthy lifestyle was the dissemination of nutrition and health knowledge through school curricula. The respondents emphasized the need to design nutrition and health-related courses for school children as well as making these courses mandatory in Indian schools. In addition, they mentioned that schools are not just meant for scholarly learning but should also serve as vital health-promotion settings for nurturing lifelong health behaviors in the pupils beginning from childhood.

*“If the matter of health is made mandatory at the school level… things like… physical exercises… or the kinds of food which need to be consumed… which… actually isn’t taught at our schools from a basic level … Now this is something which can be done. Then perhaps… we can gain (important and relevant) knowledge regarding health-related matters from an early age.”* (M29, 23 years, Kolkata)

Besides school-based nutrition and health education, the interviewees also insisted on enhancing the nutritional awareness of primary household food gatekeepers as well as the general public. They reinforced that food gatekeepers should be well-informed about nutrient composition of various food ingredients as well as meal preparation techniques to improve dietary habits at the household level.

*“I also think there should be awareness among all moms about what kinds of food to cook and the most nutritious ways to prepare them.”* (M20, 23 years, Hyderabad)

#### Creating healthy and hygienic food environments (44/79)

Another key recommendation for enabling the uptake of health behaviors proposed by the study respondents for adopting and sustaining a healthy lifestyle was creating healthy and hygienic food environments in tertiary educational institutes as well as the workplace. The interviewees emphasized the need to increase the availability and accessibility of palatable, hygienic, nutritious food in their workplace/college cafeterias in order to enhance the consumption of healthy snacks in young adults. They noted that the supply of unhealthy foods in workplace/college cafeterias should be curtailed to inhibit their consumption.

*“You see college canteen or even the hospital canteen, only oily stuff is available. Also, hygiene is compromised. They have to change everything. Proper hygiene should be maintained, and less oily and spicy food should be served.”* (M18, 27 years, Mumbai)

*“I think everyone in the college definitely goes to the canteen to munch on something and if it is a healthy snacking being it soya chips like if it is made in a tastier way but in a healthier way as well. So, it would be more convenient for us…”* (F5, 22 years, New Delhi)

## Discussion

This research inquiry contributes to the limited scholarship on healthy lifestyles in Indian young adults. The study sample conceived healthy lifestyle as consuming a healthy and balanced diet; leading an active lifestyle; and having adequate sleep. Despite depicting a thorough understanding of healthy lifestyle, the participants struggled to adopt and maintain healthy lifestyles because of three main barriers: lack of time; unhealthy food environments; and peer pressure. They proposed both individual and system-related strategies (e.g., reducing screen time) and recommendations (e.g., creating healthy and hygienic food environments) to overcome these challenges. Nonetheless, they also highlighted some important factors that supported the attainment of healthy lifestyles among young people which included intrinsic motivation, body image, family support, health promotion by famous personalities and built environment. Overall, these findings complement existing scientific evidence on healthy lifestyles in young adults across the world (9, 67–72) as well as offer novel insights from the global south.

Consuming a balanced diet, engaging in physical activity, and good sleep hygiene were perceived as important components of a healthy lifestyle in the present inquiry. Analogous perceptions of a healthy lifestyle have also been reported in Nigerian (73), American (74) and Australian (75) contexts. Interestingly, unlike their Western counterparts (75–77), none of our interviewees discussed limiting nicotine and alcohol consumption as part of a healthy lifestyle regime. This could be partly because of the prevailing stigma around alcohol consumption (78) and smoking (79) in Indian society. Another plausible reason could be that our sample was predominantly educated (i.e., two-thirds of the sample had an undergraduate degree) and local evidence suggests that tobacco and alcohol consumption is more common among the illiterate and less educated individuals (80–82).

Physical appearance, intrinsic motivation, and support from family members emerged as key enablers of healthy lifestyle during the interviews. These enablers have been cited in previous qualitative inquiries conducted in Sri Lanka (83), Australia (68), and Sweden (84) as well. Mixed thoughts were garnered regarding the impact of built environments on the uptake of healthy lifestyle in the present context. Consistent with previous evidence (34, 85–87), the majority of our sample acknowledged the positive influence of public and private gymnasiums, walking, and marathons on their lifestyles. In contrast, a couple of interviewees complained about poor infrastructure including limited or lack of green spaces, damaged gym equipment and unplanned maintenance, a barrier also echoed by males aged 30–50 years in a Mumbai-based qualitative study (36). To overcome these challenges of urban spaces, some public universities in India have created open gymnasiums within the university premises (87). These open gymnasiums have been effective in promoting physical activity among students, university staff and city dwellers (87).

The new wave of health and fitness influencers on social media was also regarded as a significant driver of healthy lifestyle by our participants. Our participants were inspired by the health recommendations of digital influencers as they tried to emulate them and their lifestyle choices for healthier living, these facilitators being widely published in the recent past (88–91). There is conflicting evidence regarding the impact of social media influencers on health behaviors and outcomes (89, 92). In view of this, policymakers should leverage the positive health impact of the influencers, while curbing the spread of misleading or detrimental health information (89, 90, 92, 93).

Recently, local government agencies in Indonesia have collaborated with social media influencers for effective public health messaging (94, 95). For example, the Makassar City Health Office in Indonesia partnered with local influencers to promote tobacco control through educational videos on social media (94). This government-influencer partnership was effective in amplifying anti-smoking messages, increasing youth engagement, and reinforcing healthy behavioral norms within the local context (94).

One of the most frequently cited barriers to adopting a healthy lifestyle among our participants were time constraints due to excessive workload or academic load. This deterred several of our respondents from engaging in physical activity or healthy meal preparation, a deterrent extensively described in the literature (34, 68, 96, 97). Considering this, time management training could be recommended for young adults to improve their study-life or work-life balance, thereby facilitating the adoption of healthy lifestyles (75). At present, medical colleges in India offer time management training as part of a one-month foundation course “Competency-based Medical Education” for undergraduate students. Perhaps, non-medical higher education institutions in India could emulate this initiative for the betterment of their students (98). In addition, workplaces could also impart time management skills to their employees as part of the induction process. Several studies have shown that time management training offered in both universities (99) and worksites (100) have proved fruitful for students’ and employees’ well-being.

Another common deterrent to adopting a healthy lifestyle was the wide availability of unhealthy food and limited access to nutritious food in the workplace, residential neighborhood, and tertiary education settings. Similar criticism of food environments has been reported previously by researchers in the global north (101, 102) and the global south (103, 104) researchers. Our interviewees also blamed the lucrative offers (e.g., direct discounts, free delivery, rewards) provided by online food delivery services for unhealthy food. This finding aligns with existing scholarship which reveals that the majority of the marketing techniques adopted by online food delivery services promote less healthy food (105–108). In fact, the overwhelming prevalence of price promotions on unhealthy foods was found to be highly appealing which influenced young consumers to make frequent unhealthy food purchases on online food delivery platforms (105, 107–109).

Several public health strategies and policies with monitoring and review mechanisms could be envisioned which may alleviate overexposure and increased accessibility to unhealthy foods available in both offline and digital food environments (108). One such strategy could be improving the healthiness of physical food environments as proposed by our young interviewees. This could be achieved by increasing the availability and visibility of healthy foods, reducing the price of healthy foods, increasing the ratio of available healthy food offerings relative to unhealthy food offerings and establishing standards for nutritional quality of the food served in the restaurants (105, 107, 108, 110). Upgrading the offline food environment will result in parallel improvements in digital food environments as both these food environments are often overlapping (108).

There is a clear absence of nutrition and food-related policies governing food environments in and around higher education institutions and worksites in India (103, 111). Therefore, the Indian Government might consider adopting the European Union’s “Digital Services Act” to ensure more clarity around food and beverage marketing through digital platforms as well as prohibit certain types of targeted marketing (110). Another feasible policy option would be the “Healthier Dining Program” implemented in Singapore which forces the food companies that operate only in digital mode to have their food and beverage items listed on the menu endorsed by the Singapore Health Promotion Board (112).

Besides improving food environments, school-based health and nutrition education was also recommended by our participants for improving lifestyles of individuals from an early age. However, the current Indian school curriculum does not prioritize nutrition and health education (113–117). Moreover, nutrition and health are mostly discussed in elective subjects like Biology and Home Science (i.e., Home Economics or Family and Consumer Sciences) and Home Science is mostly restricted to girls’ schools only (113–115). A substantial body of evidence suggests that skills-based health and nutrition programs carried out in schools have been successful in fostering healthy lifestyle and enhancing food and health literacy among school pupils (118–121). In addition, the study participants also endorsed the need for equipping Indian household food gatekeepers with adequate knowledge regarding healthy cooking practices and diet to improve lifestyle at the household level, a recommendation also reinforced by a local qualitative study (122).

A popular individual level healthy lifestyle approach adopted by the present interviewees was reducing screen time. This was effective in reducing the frequency of headaches and improving mental health as reported by our interviewees. Certainly, elevated screen time has been associated with mental health complications including anxiety and depression (123, 124) as well as migraine in young adults (125). Mirroring the views of the interviewees in the present study, a randomized control trial confirmed that three weeks of smartphone screen time reduction resulted in improvements in stress, sleep quality, depressive symptoms, and well-being in the intervention group compared to the control group (126). Therefore, similar interventions targeting reduced screen time in Indian young adults are urgently required to offset the negative health effects of excessive exposure screen time and social media addiction in them (127, 128).

### Strengths

The present research inquiry has several strengths. Using qualitative in-depth interviews, this inquiry is one of the first to capture young adults’ perceptions of healthy lifestyles in India. It provides rich insights into the factors influencing adoption and maintenance of healthy lifestyles as well as recommendations and strategies for promoting healthy lifestyles among young Indian consumers. Another major strength was the use of a multi-center approach (i.e., recruitment of participants from eight metropolitan cities in India), thus enhancing representation and strengthening transferability of our findings.

### Limitations

There are limitations in this inquiry that should be acknowledged. First, the study was primarily restricted to urban settings and therefore prospective studies could be undertaken to capture the insights of young adults residing in rural areas. Second, in spite of our efforts to recruit young adults from diverse socio-economic backgrounds, the study sample was predominantly represented by university graduates (two-thirds of the sample had attained an undergraduate degree) which could possibly limit the transferability of the present findings to illiterate or less educated population (i.e., people who did not receive any formal education). This limitation presents an opportunity to replicate this study among less educated young adults to identify additional barriers and enablers of healthy lifestyle. Third, our sample was recruited using various social media platforms and this could have resulted in sample bias and minimized the generalizability of our results. Nonetheless, social media-based recruitment methods have more potential in augmenting the reach of the study, and representativeness of the study sample, when compared to conventional recruitment strategies like telephone and flyers (129–131), thereby minimizing the possibility of any sample bias. Also, social media-based recruitment has been recognized as one of the most cost-effective strategies of sample recruitment in a brief period (129, 132). Fourth, the generalizability of the study outcomes could be limited by social desirability bias as only young adults with a strong interest in health and fitness would have volunteered for the interviews. Also, some responses might have reflected socially laudable attitudes rather than actual behaviors. Nevertheless, some interviewees candidly discussed their challenges in maintaining a healthy lifestyle as well as openly criticized the retail food environment, suggesting that socially desirable responses were minimal in the present context.

## Conclusion

This qualitative inquiry provided unique insights on how Indian young adults perceive healthy lifestyle and the facilitators associated with the attainment and maintenance of healthy lifestyle. Additionally, the participants discussed the various barriers, notably time constraints and unhealthy food environments in university and workplace settings, which inhibited them from adopting healthy lifestyle. Finally, they shared several strategies and recommendations to counteract these deterrents. This first-hand information could be channelized towards the development and implementation of tailored behavioral interventions aimed at improving lifestyle as well as overall health of urban Indian young adults.

## Data Availability

The raw data supporting the conclusions of this article will be made available by the authors, without undue reservation.
